# When procrastination pays off: Role of knowledge sharing ability, autonomous motivation, and task involvement for employee creativity

**DOI:** 10.1016/j.heliyon.2023.e19398

**Published:** 2023-09-11

**Authors:** Ahmad Adeel, Samad Sarminah, Li Jie, Daisy Mui Hung Kee, Yahya Qasim Daghriri, Rsha Ali Alghafes

**Affiliations:** a Department of Business Education, The University of Chenab, Gujrat, Punjab, Pakistan; b Department of Business Administration, College of Business and Administration, Princess Nourah Bint Abdulrahman University, Riyadh, 11671, Saudi Arabia; c College of Economics & Management, Hubei University of Arts and Science, Hubei, China; d School of Management, Universiti Sains Malaysia, Malaysia; e College of Business and Administration, Princess Nourah Bint Abdulrahman University, Riyadh, 11671, Saudi Arabia

**Keywords:** Procrastination, Knowledge absorption, Task engagement, Autonomous motivation, Creativity, Self-determination theory

## Abstract

The prime objective of this research was to investigate procrastination as a prospectively constructive element of the creative process among employees working at different hierarchical levels in a Chinese organization. Building on self-determination theory, this research postulates a connection between procrastination and creativity through the incubation of knowledge absorption, autonomous motivation and task engagement as boundary conditions. Data was collected from 213 individuals from the workforce and their immediate managers belonging to a Chinese furniture company; then analyzed with Mplus for simple regression analysis, mediated moderated analyses, and coefficient estimates of all the study variables. The outcomes of this investigation showed an inverse relationship between procrastination with creativity, while creativity being strongest in the medium levels of procrastination; however, when autonomous motivation and/or task engagement are strong, procrastination depicts an inverted-U-shaped association; however, in scenarios where both autonomous motivation and the task engagement are low, procrastination has a negative linear relationship. With the results of this research, we have shown that moderate procrastination has a causal effect on the generation of creative ideas. This research demonstrated that as long as employees had strong autonomous drive or high task engagement, their supervisors awarded them better ratings when they procrastinated moderately on their assignments. Limitations and future research directions were also discussed.

## 1 Introduction

People frequently delay things consistently [1,2]. According to studies, chronic procrastination has impacted almost 20% of the population overall [3]. Also, around 80–95% of college students accept being tilted toward procrastination [4,5]. In environments with constrained obligations to meet specific timelines, such as workplaces, procrastination tendencies have been especially observed [6,7]. A generalized perspective of procrastination relates it to counter-productivity [[7], [8], [9]]. Procrastination can also be taken as an inability to self-regulate [10,11], which in turn leads to compromised work efficiency and individual objectives, requiring measures and efforts to cope with [4]. Since reduced work performance outcomes are found to be relevant to procrastination, prevailing data confirms this observation on empirical grounds [12], as well as compromised performance in academics [13,14], along with adverse impact on financial aspects including but not limited to tax overpayments [15].

Experts have generally ignored the likelihood that procrastination could be beneficial by concentrating mostly on the adverse effects of the behavior. One attribute related to performance that works on varying sets of predictors as well as techniques as compared to work satisfaction [16] or production efficiency [17] is creativity, which may appropriately be described as the process of generating innovative perspectives as well as practical insights [18,19]. Such an approach raises the likelihood that procrastination may not have the same negative effects on creativity as it impacts task productivity or efficiency. In two laboratory experiments, the researchers have found that procrastination is not always harmful to the generation of creative ideas; these findings are strengthened by the field study with a sample of 170 employees; the supervisors rate higher on the creativity scale only to those employees who procrastinate for generation of novel and useful ideas [20]. Additionally, In the early 2000s, China reported its first-time double-digit gross domestic product (GDP), and the economy has been rapidly growing since then; one of the main contributors is the potential of Chinese firms to innovate in an ever-changing corporate world [21,22]. On the other end, the researchers have also reported that the tendency to procrastinate among young Chinese adults is increasing [23]. Therefore, investigating the relationship between procrastination and creativity in a Chinese context is significant for Management. Thereby, this research investigates the notion that, by promoting incubation, procrastination within moderation may improve creativity within the rapidly developing economy of China.

Procrastination may prove to be beneficial for creative ability; according to some experts, procrastination may contribute to improved work efficiencies in creative activities or challenges that demand an inquisitive requirement of knowledge [24] and may contribute to attaining favorable results for professionals in jobs demanding innovative approach [25]. As elaborated by these researchers, the time whiled away procrastinating may not be entirely lost because it can result in an unanticipated gain on the postponed activity. Procrastination may prove to be a way of availing a bit of surplus time in which new insights might be learned [17]. Additionally, individuals who were actively involved in creative work exhibited a recurrent tendency of procrastination, as proposed by research by Westinghouse Talent Search award-winners regarding adulthood on qualitative grounds [26]. According to anecdotal evidence, great and famous personalities belonging to diverse professions, like Leonardo da Vinci, were chronic procrastinators [27], and procrastination by Albert Einstein, Frank Lloyd Wright and Thomas Edison is also known to have occurred [28]. Some such famous personalities like writer Margaret Atwood have admitted to their tendency towards procrastination. She observed that she usually whiled away the start of the day procrastinating accompanied by worrying, eventually plunging into her writing, frantically nervous and anxious, way past noon [29], despite the fact she has been outstanding as a writer by being able to publish around 60 books, which include her best sellers like “The Handmaid's Tale,” and received multiple awards for her originality. Although these intellectual suppositions and anecdotal evidence are indicative of the fact that there could be circumstances in which procrastination fosters creativity, these assumptions have not yet been fully established or experimentally tested.

In this research, novel perspectives explore the correlation of procrastination with creativity, along with the intermediary impact of incubation processes accompanied by the moderating impact of boundary conditions. Though a linear relationship between procrastination and creativity has been observed and proposed by creativity intellects, this paper contends that this relationship is curvilinear, inverted-U shaped, under the mediation impact of incubation processes, in combination with moderation by motivational drive and task engagement. The study involves scrutiny of the extent to which procrastination may encourage the restructuring of problematic concerns as well as the stimulation of novel intuitions by reflecting on concepts of incubation and temporality. Individuals who don't procrastinate run the danger of overlooking pretty simple first possibilities because the first few thoughts that strike someone's mind are usually one of their most typical ones [20,30]. Workers who procrastinate moderately have more time for incubation, which enables them to tackle the issue from new angles and obtain remote understanding and insight. Nevertheless, where there is an increased tendency of individuals to procrastinate a lot, there will be a higher probability of defining the goal precisely and conclusively [31] and feel pressure to address the issue right away in the scenario where they actually start making progress [32,33]. Researchers taking these perspectives have unambiguously emphasized the criticality of autonomous motivation in comparison to controlled motivation when employee performance under investigation is discretionary as well as complex in nature [34]. Thus, our focus will remain on autonomous motivation while investigating the procrastination and creativity of employees. It is also anticipated that people are prone to be more creative in their approaches when they possess the motivation or chance to do so. This is only possible given that employees have a purpose and motivation for being aware of their objectives intuitively while practicing procrastination under certain boundary conditions.

**Fig. 1:**
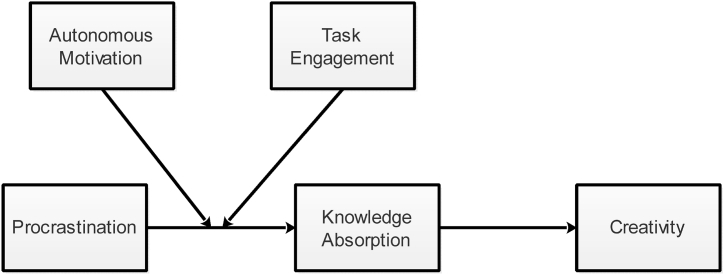
Conceptual model.

These proposed relationships have been investigated based on research data regarding procrastination exhibited by the workforce alongside ratings regarding creativity from supervisors' perspectives, the mediating process of knowledge absorption, where the boundary conditions of autonomous motivation and task engagement were put to trial, which resulted in confirmation of the curvilinear relationship amongst procrastination and creativity. The available evidence on time management, creativity, and motivation has benefited greatly from the theoretical viewpoints and empirical findings of this study. The outcomes of this contradict and put to trial the widely held belief that procrastinating is always counterproductive, showing that while it reduces work efficiency if practiced moderately, procrastination can actually increase creativity. By this approach, it is possible to respond to needs for novel perspectives catering to the behavioral antecedents of creativity [35] as well as general curvilinear relationships by identifying acts of moderate procrastination to be an initiative on individual levels which may lead to enhance creativity under the influence of autonomous motivation alongside potentials to come up with fresher perspectives of problem-solving [36,37]. In achieving the core objective, this study proposes to normalize procrastination as a prospectively constructive element of the creative process in certain scenarios, such as when the delays are reasonable, the issue is intriguing, or a fresh approach is required. In the next sections, we will develop hypotheses based on the relevant theories and literature available. After that, with the help of relevant measures, we will collect data from employees of a Chinese organization, and then we will analyze our data with Mplus for simple regression analysis, mediated moderated analyses, and coefficient estimates of all the study variables. We will then explain our results. Explain theoretical contributions, practical contributions, and conclude our research paper by explaining limitations and future research directions. Proposed relationships are represented in Fig. 1.

## 2 Literature review and hypotheses development

### 2.1 Procrastination and creativity

Procrastination is defined as the act of purposefully delaying task progress or completion of a task while being aware of the fact that it may come at a cost [38,39], while creativity is defined as the generation of novel and useful ideas [40]. Two important characteristics set procrastination apart from other types of delay [28]. In the first place, delaying a task is a choice, as opposed to being compelled to do so by external circumstances like a computer malfunction or a delayed trip. It also entails delaying the beginning, progressing, or end of activity while being conscious that doing so might have drawbacks. It's vital to distinguish between the concept of procrastination and its assumed effects, even if some historical interpretations of procrastination have included this idea [41]. It is crucial to conceptually construct and actually examine the effects of procrastination instead of relying on mere presumptions of it being invariably problematic.

Procrastination doesn't have, in every case, adverse implications [42]. “Procrastinate,” a Latin verb, which means to put off or delay till some other day, is where the term “procrastination” originates [43,44]. The Latin prefix “pro,” here signifies being forward or being in approval of, and the word “crastinus,” expresses a reference to tomorrow, are combined to form this term [45]. This indicates that there isn't a portion of the term that is connected to a negative connotation. There are two verbs from ancient Egypt that may be interpreted as “to procrastinate,” one of which refers to lethargy towards completing important chores and the other of which refers to the good strategy for avoiding redundant exertion in addition to impetuous labor [46]. Procrastination was also employed by the Romans to describe sophisticated decision-making over when not to act, like understanding the point to hold off until the ideal moment to implement a military tactic [2]. Such traditional meanings addressing the root word provide more evidence that procrastination was not always considered a negative practice. Therefore, certain modern intellects have claimed that procrastination's adverse implications didn't develop until after the Industrial Revolution; alongside growing technology, effectiveness and timeline compliance became more crucial [47]. Therefore, it is plausible that procrastination may have unanticipated gains in scenarios where efficiency is not the prime objective [48].

The core focus of this study is on the part procrastination plays in the conception of innovative and beneficial perspectives [19]. Instead of assessing, growing, or applying such ideas, this study particularly focuses on exploring procrastination as a prospectively constructive element of the creative process. Individuals that procrastinate are postponing required action for a job that seems costly. In the meantime, the problem's solution and intended response may be running through their subconscious thinking. Procrastinating moderately might boost creativity by enabling the cultivation processes addressing issue rearrangement alongside stimulation of innovative perceptions, reflecting on theories of incubation and time. Knowledge absorption is defined as rearranging and reshaping one's cognitive understanding of the relevant task [49]. The exploration of new information or the retrieval of previously stored information that was previously inaccessible is both necessary for the activation and absorption of new knowledge [50,51]. According to researchers, solving an issue creatively might result from looking at it from a fresh angle and combining background knowledge [52].

### 2.2 Mediation of knowledge absorption

It is suggested that procrastination affects creativity in the presence of some incubation. When workers don't put off starting a job at all, they begin working; they engage in the task as soon as it gets available, hence preventing issue reorganization as well as the stimulation of new information. According to recent research, idea development frequently starts with very apparent concepts [53], which might limit further inventiveness [54]. Individuals may have an innate inclination towards linear thought process, which causes them to reach a point of seizing and freezing on an initial traditional thought if they are stuck on the way the task challenge was first framed and have accessibility to just easily available knowledge [52,55]. As Rosenbaum, Gong [56] noted, individuals frequently procrastinate or take the ‘low-hanging fruit’ before beginning work [57,58]. This is often referred to as engaging with a prejudiced perspective, as individuals cannot fully grasp the issue and consider a variety of potential remedies before choosing a single to apply [59]. According to the latest study, people who postpone also have trouble managing their time, which may explain why they have trouble finishing projects promptly even when they do not procrastinate as often [60]. According to previous studies on the Einstellung effect (problem-solving), individuals frequently adopt the same strategy for an issue or set of problems, indifferent to the fact that the opted strategy is effective [61]. Thus, the lack of procrastination can severely restrict incubation, leading to a less creative result.

On the other hand, workers who procrastinate in moderation begin working on the assignment fairly late, near the mid of the timeframe. Advancing without the core focus on the task's initiation and its deadline may help with issue sorting and activating innovative insights, as per studies regarding the psychology of time. In order to increase the extent of abstract thought ability concerning the what and whys of the task, instead of concentrating specifically on finding ways to its solution, moderate procrastination separates oneself from the problem psychologically, enabling separation both temporally as well as spatially [20,39].

Individuals can consider the work challenge without being constrained by the initial framing as well as the significance of the deadline by engaging in mild procrastination. They are prone to transcend the prevailing problem structure [62] when their thoughts drift to seek novel and unconventional perspectives on a challenge [63]. After all, according to researchers, a period of disengagement regarding a specific work enables an individual to tackle such a situation again by revisiting/resuming it, which also, perhaps with a diverse and fresher insight and better grasp, serves as a window for innovative insights and knowledge [16]. As a result, they have the chance to reconsider the issue and consider a variety of alternative remedies, both consciously and unconsciously [64]. So, in as much as it allows workers to unconsciously or consciously consider other approaches to the issue, moderate procrastination can help workers come up with more original solutions.

Additionally, the stimulation of new perspectives is also fostered by moderate procrastination. Individuals who procrastinate moderately are less likely to commit to actively resolving the issue [64], which weakens goal shielding [65] and increases the likelihood of consultation of remote knowledge. Workers are prone to make unanticipated jumps to less clearly pertinent information when their focus is off-target [66,67]. Researchers also discovered in qualitative research that early-career researchers aided from procrastination in the form of an incubation period to avoid any possible preterm selection of research topic or explanation [26]. It was put forth by one of the scientists that ideas need time to grow in scientific endeavors. I am frequently spontaneous; hence my instincts have been erroneous. Therefore, the speaker said I procrastinate to refrain from responding too early. The cognitive accessibility of remote knowledge and innovative information may have been what these scientists were waiting for. Collectively, these considerations show that moderate procrastination aids workers in striking a creative proportion between activities that foster creativity and those that seek closure.

Nevertheless, excessive procrastination has the least likelihood of having the same positive effects as moderate postponement. High procrastination will likely limit incubation, and this expectation can be justified on both a cognitive and an emotional level. Individuals inclined towards excessive procrastination commence with the assignment only after deadlines start to close. Incubation is automatically limited when advancement starts near to deadline. According to cognitive theory, at such a time, incubation processes would be hampered both consciously and subconsciously owing to the pressure of aim completion [68]. Since the conscious focus is on coming up with an instant fix instead of the most creative answer, workers are inclined to skip conscious incubation. As a result, they adopt an implementation mentality instead of a deliberation attitude [69]; hence, they are more inclined to spend less time reorganizing the issue and considering innovative perspectives. Such people are inclined to lose out on subconscious incubation as they rush to find a solution since they need a pause or a diversion to give those processes a chance to work [16]. Such attitude restricts the workers from unlocking the grassroots, diverse, high-capability processing regarding intuitive incubation, confining to hierarchic, converging processing which is limited to the current issue premises only accompanied by the knowledge which is pertinent on its own [70,71].

Additionally, when people procrastinate for more time, the resulting temporal urgency is probably going to prevent incubation since it will force them to see the issue more practically instead of abstractly. According to research, persistent procrastinators prefer to view activities in a definitive manner [72]. Additionally, studies have demonstrated that temporal acuity might limit creativity by encouraging a more literal approach in place of abstract interpretations [73]. Workers tend to be more concerned with the specificities of finding ways to come up with a solution to the issue as the task's deadline draws nearer [74]. Also, they exhibit lesser tilt towards opting for the abstract conceptions frequently required for the discovery of novel approaches to structure any problematic areas while incorporating further distant as well as varied origins of knowledge. Individuals who procrastinate somewhat have an adequate temporal edge to conceptualize the work on an abstract basis [75], which enables one to consider various problem-solving options and stimulate new information.

From an affective standpoint, high procrastinators' frame of mind is prone to obstruct incubation by the time they begin thinking about the job. The saying good things satiate and bad things escalate has been around for a while [76]. As tendencies of people to procrastinate increases, this causes delays in the incubation process and more, the expense of time pressure might increase. When workers put off tackling a subject later on, the resulting time pressure might cause focus and attention to become more narrow, which, in turn, has been experimentally related to causing lowered creativity [77]. Goal shielding, which blocks out information that doesn't seem to be necessary to finish the job, is likely to be activated by high levels of procrastination [6,31,43,48]. The desire to find a quick fix to the situation at hand [30] may cause employees to become overly inflexible, focusing on tried-and-true methods rather than indulging in trying fresher perspectives [78]. High procrastinators are prone to become distracted by the minutiae of execution rather than being excited about the mere potential that lies within innovation [79]. Thus, excessive procrastination possibly limits incubation, which would impede innovation. By relieving workers of the restrictions of job accomplishment objectives, tangible analysis, time constraint, and threat-rigidity, moderate procrastination, in contrast, is likely to foster creative incubation. They have the freedom to consciously and unconsciously reconstruct the challenge and trigger fresh information that is remote from the standard framing of the work in the absence of pace problems and impending deadlines.

H1 There exists a curvilinear correlation between procrastination and creativity.

H2 The curvilinear correlation of procrastination with creativity is mediated by knowledge absorption.

### 2.3 Task and motivational moderators

Procrastination's curved impact on creativity is subject to crucial boundary circumstances. This study uses theories of self-determination [80] and motivational equifinality to investigate such instances. Though moderate procrastination may offer workers the chance to incubate, this work predicts the possibility of the workers taking use of such a chance to foster creativity entirely relies on the job assigned as well as the degree of their drive. More precisely, it is predicted that the curvilinear impact of procrastination upon creativity will have a higher likelihood to manifest itself in scenarios where workers' involvement in their tasks is evident or in scenarios where workers are autonomously motivated (or self-motivated), defined as the motivation of people engaging in an activity with a full sense of willingness, volition, and choice [80,81].

Motivation represents the urge to perform a job out of satisfaction with pleasure [19,82]. Self-determination theory's basic tenet states that workers are psychologically driven; they are lured to completing tasks, the reason being they find them fulfilling as well as contenting [83]. Self-determination theory elaborates that individuals can be motivated autonomously and also controlled [54]. When employees are autonomously motivated, their engagement in an activity and their conduct are self-validated and harmonized as per their personal inclinations as well as ideals [34,84] as compared to controlled motivation when employees feel under pressure originated from external pressure or self-imposed pressure [85].

According to statistics, people indulge in work with greater interest, cognitive adaptability, and effort, accompanied by tenacity, given that they feel genuinely driven [86]. Even though decreased procrastination has been scientifically related to motivation, there is not a strong correlation between these factors [5]. Provided the condition of challenging or intimidating responsibilities [26], or whenever tasks with more interest arise, autonomously motivated workers might well procrastinate completing the work at hand. Autonomous motivation may vary depending on the nature of the jobs as well as with time [20]. Resultantly, although it is not anticipated that autonomous motivation may invariably stop procrastination, rather it can be anticipated that it impacts the factors that occur when the prime focus is diverted from the required area.

According to theory, one of the motivation's core roles is to focus attention [87]. The degree of autonomous motivation that individuals have during procrastination on moderate levels is deterministic of the fact whether full attention is laid on the primary goal or only to a portion of it. Workers lacking autonomous motivation may, contradictorily, consider the time spent while away from the job as an escape from all labor. The disinterest in the focal area is mistaken as a cue to completely halt the thought process on the primary goal [88]. However, self-determination theory proposes that if workers discover the task put off to be of interest to them, they have a tendency to be concerned about the ins and outs of the task while moderately procrastinating. The reason behind the interest in that specific area keeps them engaged subconsciously and intently [89], hence seeking their consideration resulting in innovation [67]. Autonomously driven workers have the inclination to put off important activities during day time, performing thoughtless ones with little effort, intensive tasks, and cognitive challenges, eventually enabling them to save their energies for incubation [16,90]. Workers who are autonomously altruistic exhibit an inclination to take challenges as opportunities instead of hardships [19]; this encourages them to continue their thought process on the required area, completely giving it a pass. This allows for structural reforms in the concerned area along with the stimulation of innovative perspectives to take place during periods of moderate procrastination.

To compensate for the absence of autonomous drive, nevertheless, the need for creativity could well be substituted by moderate procrastination. The measure of how much a worker's employment both permits and encourages the development of innovative thoughts encompass the core of task engagement [34,84], defined as “the degree to which individuals invest their physical, cognitive, and emotional energies into a specific task that composes part of their work role” [91].

For certain individuals as well as positions, creativity stands as a prime goal [32,92], while for others, it is neither anticipated nor promoted; in such environments, it may even be prohibited [37,93]. Creative endeavors generally encourage autonomous motivation [40,94]. However, it is conceivable to feel autonomously driven while creativity is not anticipated and similarly feel unmotivated wherever creativity is a requisite [87,95]. Autonomous motivation, as per the self-determination theory [85], rests on satisfying fundamental psychological reasons for self-reliance, capability, and connectedness. Individuals may exhibit motivation in environments lacking task engagement if they are given autonomy over the ins and outs of a task if they know how to cope or be in control, that also with others are supportive of them [82]. Workers' autonomous motivation has a higher likelihood of being suppressed despite the presence of creative tasks, given that they lack the liberty to opt, foster feelings of being inept, or consider themselves to be undervalued [83]. They can participate because of these differences between autonomous motivation and task engagement. Hence it is suggested that a task engagement can recompense for any deficiency of autonomous drive for enhancing the curvilinear impact of procrastination over creativity.

According to self-determination theory, there are several levels of extrinsic and intrinsic motivation that are fueled by laying emphasis on the outcomes of the job, whereas pure autonomous drive involves engaging in an activity with a definitive end in consideration [82]. Intrinsic and extrinsic motivation may be taken as an urge to put forth action in a job at hand since it is essential to a worker's evaluation as well as self-image (integrated regulation), hence fulfilling a few too many of their norms/beliefs (identified regulation), it helps the employees in feeling better about themselves or prevent unnecessary remorse (introjected regulation), or it helps in getting rewards encouragement or avert penalty (external regulation) [83]. Workers exhibit a higher likelihood of being committed to the objective of becoming creative whenever a task has significant engagement needs based on the importance placed on innovative and beneficial perspectives [92]. Despite the fact that they aren't autonomously task-driven, whenever individuals accept the intrinsic or extrinsic objective, they are inclined to be more focused on reaching the external, introjected, identifiable, or integrated benefits of putting forth creative perceptions. They possess a purpose to be creative even though they lack the energy to do it [89].

Although individuals do not possess the innate capability of being motivated workers, task engagement inspires them to keep their thought processes running subconsciously while they practice moderate procrastination [96]. There might not exist any compulsion to think about the assignment any longer, yet they will be compelled to Ref. [97]. Limited focus is laid on the core issue while simultaneously being engaged in several diverse pursuits, enabling them to structurally reform the problematic area as well as stimulation of innovative perspectives while procrastinating moderately. This partial attention is assigned to the core goal while pursuing intrapsychic, impression management, or tangible incentives affiliated with fostering creative perspectives. Undoubtedly, studies have proven that whenever a job necessitates diverse reasoning, incubation is certain to provide newer angles [98]. Individuals will be prevented from completely disassociating from the primary activity while procrastinating somewhat by creative constraints.

It is anticipated that autonomous drive and task engagement will act as replacements rather than complementary factors or boosters. Self-determination theory claims that autonomous regulation needs reasoning or motivation singularly rather than both [83]. More is not necessarily better while considering varying sorts of motivations. Hence, quality supersedes quantity [86]. Masses exhibit a tendency to be independently motivated given a task is engaging (and therefore autonomously motivating) or individually significant (and so independently intrinsically or extrinsically motivating). Motivational equifinality is the situation in which one objective is achieved by numerous replaceable approaches [99]. Without the apparent merger of means and ends, which makes an action its own objective [99,100], considering the ends as valuable functions as a stand-in for autonomous drive [101,102]. In fact, studies have shown that a situation that supports a behavior, as well as an internal drive for that activity, can serve as a replacement [102,103]. For moderate procrastinators, the autonomous drive will lead to incubation when task engagement is absent. If there is a task engagement, even if the person is uninterested in the problem-solving techniques, they are likely to be worried enough about the result to maintain the work in their subconscious, allowing for a fresh viewpoint on the situation and the application of new information. These arguments suggest that when autonomous motivation and/or task engagement are present, moderate procrastination can encourage inventiveness.

H3 The curvilinear correlation of procrastination with creativity is contingent jointly on autonomous motivation and task engagement.

## 3 Methodology

In this study, 213 individuals from the workforce, along with their respective managers at a Chinese furniture company, were surveyed. In 2022, the Chinese furniture industry earned a profit of almost 34.42 billion Yuan, 3.8% over 2021 (Chinese national bureau of Statistics, 2022). Managers' feedback revealed spontaneous variability in the core ideas of procrastination and creativity. This firm has a work experience of a diverse nature in furniture-related fields, which provided a variety of time-sensitive situations and opportunities for coming up with unique and worthwhile solutions to problems. The collected data were then analyzed with Mplus and then analyzed with Mplus for simple regression analysis, mediated moderated analyses, and coefficient estimates of all the study variables. We have also added VIF (Variance Inflation Factor)- a measure of multicollinearity among the independent variables in a multiple regression model. The results are provided in Table 5. The VIF values range from 1.082 to 1.674, with a tolerance level ranging from 0.597 to 0.924; indicating that the variables are moderately correlated (This study was approved by the Ethical Committee-The University of Chenab; Reference: 03BE23).

The business featured a wide range of divisions, namely strategic, sales division, design division, marketing division, quality control, production control, and customer service. Individual representatives from all of the departments were part of the final sample; they all worked “nine to five” jobs in a typical office setting. 286 workers from a cross-section of office positions inside the company were given the questionnaire and were asked to participate fully in private academic research concerning creativity in the workplace. The completed questionnaires were submitted by 213 workers resulting in a response rate of 74% in totality. Participants were inquired to rate their procrastination at the workplace in the questionnaire. It was requested to share feedback from the focal employee's immediate manager. The process was repeated for every worker hence resulting in a total of 213 individuals' feedback. The age bracket of workers on average was 34 years, with the addition that they had been in their present role for 6.13 years on average. With 37 supervisors, there were little over five employees per supervisor on average.

### 3.1 Measures

#### 3.1.1 Procrastination

Following previous research with personal project methodology, we measured procrastination [104]. Employees first wrote their five core tasks (an appropriate number of core tasks) assigned to them as per their job requirements and subsequently stated the degree of practiced procrastination while performing dedicated primary objectives they were held responsible for. The range of scale items was considered from 1 = not at all to 5 = to a great extent. We then average each and every task score to measure procrastination for each and every employee (α = 0.82).

#### 3.1.2 Knowledge absorption

To measure knowledge absorption capacity, employees provided their individual responses with a four-item five-point Likert-type scale [105]. Sample items include: “I may promptly judge how more useful the new information and knowledge is than the existing ones.” and “I may accept the task-related information and knowledge well.” The range of scale items was considered from 1 = strong disagreement to 5 = strong agreement (α = 0.87).

#### 3.1.3 Autonomous motivation

To measure autonomous motivation, employees provided their individual responses for the Self-Regulation Survey [106]. From the perspective of current research, motivation represents motivation specific to the area (situational motivation) of absorption of knowledge. Using Self-Regulation Survey [106] to estimate autonomous motivation and the extent of intrinsic as well as identified motivation, we asked the respondents about any inherent causes/motives for engagement in knowledge absorption. Previous researchers have also used this technique while measuring autonomous motivation using SRQ [107]. Employees were asked, “Why do you absorb knowledge?” And given the following four items: “… because I enjoy it” (intrinsic), “… because I like it” (intrinsic), “… because I find it personally satisfying” (identified), and “… because I think it is an important part of my job” (identified). The paradigm of autonomous motivation was then created with an average of these four items' responses [107]. The range of scale items was considered from 1 = strong disagreement to 5 = strong agreement (α = 0.78).

#### 3.1.4 Task engagement

We measured task engagement with nine items five-point Likert-type scale [108,109]. The employees were asked to exhibit behaviors that are indicative of the level of task engagement. Sample items include “I exerted my full effort in the task” and “I felt energetic working on the task,” The range of scale items was considered from 1 = strong disagreement to 5 = strong agreement (α = 0.82).

#### 3.1.5 Creativity

Supervisors rated each and every employee working under their supervision with three items, five points Likert-type scale [110]. A sample item includes “creating new ideas for improvements.” The range of scale items was considered from 1 = by no means to 5 = highly probable (α = 0.83).

#### 3.1.6 Control variables

For the current research accounted for gender, age, education, and job tenure. Employee motivation may impact creativity as well [111]. Therefore, employees' intrinsic motivation and extrinsic motivation were also accounted for during the research. We measured both types of motivations by inquiring individuals along the lines of: “Why are you motivated to do your work?” Sample-item catering Intrinsic motivation constitutes: “Because I enjoy the work itself” (α = 0.81), and the sample item for extrinsic motivation is: “Because I need to earn money. (α = 0.86). The range of scale items was considered from 1 = strong disagreement to 5 = strong agreement.

## 4 Results

Table 1 gives basic statistical computations, including means, standard deviations, and correlations for the variables under consideration for the survey. In this research, we performed simple regression analysis, mediated moderated analyses, and coefficient estimates of all the study variables with Mplus. These results are presented in Table 2, Table 3, Table 4, respectively. The study drew a comparison amongst those who responded and those who didn't on the demographic factors of age, sexual-category, along-with literacy in order to evaluate non-response bias [112]. On any of these factors, there were no noteworthy variations. The predictor variables that constitute procrastination, autonomous motivation, task engagement, extrinsic motivation, intrinsic motivation, age, sexual category, literacy, along-with job tenure were standardized before analysis. As the data was nested, therefore random coefficient modeling was used to adjust for it. Table 4-Step 3 and 4 displays regression models that include procrastination, procrastination squared, and the factors that impact creativity. The findings indicate that procrastination and creativity are inversely correlated, with creativity being strongest in the medium levels of procrastination (Fig. 5). Each increment of procrastination diminishes creativity while being within the moderate-high range of procrastination, while it improves it for the low-moderate range of procrastination. Therefore, creativity increases as one approaches moderate procrastination from either range of extremities procrastination.

**Table 1:** Means, Standard Deviation, and Correlation among study variables.

Variable	Mean	SD	1	2	3	4	5	6	7	8	9	10
1. Gender	0.53	0.31										
2. Age	26.73	3.25	0.202									
3. Education	2.42	0.61	0.236	0.286								
4. Job tenure	6.13	2.93	0.327	0.261	−0.213							
5. Intrinsic motivation	3.28	0.26	0.265*	0.273	0.267	0.286						
6. Extrinsic motivation	3.97	0.29	−0.235	−0.297*	−0.103	0.234*	0.324*					
7. Procrastination	2.63	1.06	0.312	0.264	0.329	0.423*	0.232	0.284				
8. Knowledge absorption	4.39	0.85	0.329	0.321	0.234*	0.124	0.125	0.343	−0.453			
9. Autonomous motivation	4.09	0.65	0.083	0.218*	0.257	0.242	0.012*	−0.432	0.546	0.532*		
10. Task engagement	4.08	0.66	0.238*	0.383	0.129	0.233*	0.234	0.432*	0.426	0.238	0.501*	
11. Employee creativity	3.93	0.74	0.273	0.085	0.092	0.252	0.357	0.462	−0.352*	0.432	0.364	0.238

*Note. Observations* = 213. Gender was coded as 0 = Female, 1 = Male. Education was coded as 1 = College Graduate, 2 = Bachelor Degree, 3 = Master Degree.

*p < .05. **p < .01.

**Table 2:** Regression analyses.

Predictor	Model 1		Model 2		Model 3		Model 4	
	Knowledge Absorption		Knowledge Absorption		Knowledge Absorption		Knowledge Absorption	
	Estimate	*T*	Estimate	*t*	Estimate	*t*	Estimate	*t*
**Control Variables**								
Gender	0.216	1.271	0.237	1.248	0.575	1.755	0.254	1.454
Age	−0.471	1.242	−0.293	1.234	−0.475	1.573	−0.237	1.345
Education	−0.237	1.362	−0.482	1.325	−0.647	1.765	−0.287	1.346
Job tenure	0.023*	1.997	0.097	1.598	0.897	1.345	0.097	1.862
Intrinsic motivation	0.673	1.878	0.237	0.234	0.878	0.573	0.877	0.878
Extrinsic motivation	0.785*	1.974	0.537	0.656	0.455	0.536	0.757	0.758
**Independent Variable**								
Procrastination	0.139*	2.102	−0.537	1.209	−0.229	0.978	−0.258	0.114
**Moderating Variable**								
Autonomous motivation			0.478	1.679			0.049	0.986
Task engagement					0.289	1.209	0.010	1.016
**Interaction Effects**								
Procrastination X Autonomous motivation			0.137*	2.181			0.419*	1.991
Procrastination X Task engagement					0.056*	2.267	0.313	1.678
Autonomous motivation X Task engagement							−0.026	1.235
Procrastination X Autonomous motivation X Task engagement							0.084*	2.325
Δ *χ* 2 (Δdf)	35.14(7)**		37.65(7)**		39.64(12)**		42.75(14)**	
Δ *R*^*2*^	0.35		0.39		0.47		0.61	

*Note. Observations* = 213. Gender was coded as 0 = Female, 1 = Male. Education was coded as 1 = College Graduate, 2 = Bachelor Degree, 3 = Master Degree.

*p < .05. **p < .01.

**Table 3:** Mediated moderation analysis results.

Predictor	Model 1		Model 2		Model 3	
	Employee Creativity		Knowledge Absorption		Employee Creativity	
	Estimate	*t*	Estimate	*t*	Estimate	*t*
**Independent Variable**						
Procrastination	0.139*	2.051	0.141*	2.13	0.14	1.71
Δ *R*^*2*^	0.02*		0.02*		0.01	
**Moderating Variable**						
Autonomous motivation	0.12	1.59	0.06	1.03	0.07	1.14
Task engagement	−0.11	−1.41	−0.12	−1.39	−0.05	−0.71
Δ *R*^*2*^	0.01		0.01		0.01	
**Interaction Effects**						
Procrastination × Autonomous motivation	0.17	2.09*	0.14	2.21*	0.06	0.91
Procrastination × Task engagement	0.22	2.58**	0.16	2.39**	0.11	1.39
Procrastination × Autonomous motivation × Task engagement			0.16	2.11*		
Δ *R*^*2*^	0.02*		0.02*		0.01	
**Mediating Variable**						
Knowledge Absorption					0.54	8.61***
Δ *R*^*2*^					0.32***	
**Interaction Effects**						
Knowledge Absorption × Autonomous motivation					0.03	−0.34
Knowledge Absorption × Task engagement					−0.08	−1.03
Δ *R*^*2*^					0.01	
Δ *χ* 2 (Δdf)	28.24 (7)**		32.65 (11)**		34.65 (9)**	
Δ *R*^*2*^	0.47		0.43		0.41	

*Note. Observations* = 213. Gender was coded as 0 = Female, 1 = Male. Education was coded as 1 = College Graduate, 2 = Bachelor Degree, 3 = Master Degree.

*p < .05. **p < .01.

**Table 4:** Coefficient Estimates of all the variables.

Variables	Step 1: Control Variables			Step 2: Control Variables and Procrastination			Step 3: Curvilinear effect of procrastination			Step 4: Two-way interaction effects			Step 5: Quadratic three-way interaction effect		
	γ	SE	T	γ	SE	t	γ	SE	t	γ	SE	T	γ	SE	T
Intercept	4.16	.12	36.13**	4.19	.12	36.32**	4.31	.11	35.45**	4.31	.11	36.98**	4.37	.11	36.79**
Autonomous Motivation	.07	.07	.95	.07	.09	.79	.09	.09	.95	.06	.11	.46	.05	.11	.60
Task Engagement	.06	.09	.64	.05	.09	.51	.04	.07	.42	.17	.09	1.61	.16	.09	1.71
Gender	.01	.08	−.08	−.01	.08	−.26	−.02	.06	−.51	−.06	.08	−.71	−.03	.06	−.63
Age	.04	.12	.26	.02	.12	.33	−.02	.10	−.07	−.04	.12	−.24	−.05	.10	−.56
Education	−.26	.10	−3.09**	−.26	.08	−3.21**	−.30	.08	−3.56**	−.35	.09	−3.91**	−.32	.09	−4.05**
Job tenure	.01	.08	.07	.02	.06	.09	−.01	.06	−.32	−.05	.08	−.61	−.04	.06	−.51
Intrinsic motivation	.06	.11	.54	.06	.11	.54	.10	.11	.100	.11	.09	1.24	.15	.09	1.66
Extrinsic Motivation	.04	.12	.55	.04	.11	.56	.11	.11	.100	.11	.09	1.25	.15	.11	1.66
Procrastination				−.11	.07	−1.59	−.03	.06	−.36	−.02	.07	−.39	−.02	.09	−.14
Procrastination^2^							−.11	.06	−2.29	−.13	.06	−2.70	−.21	.08	−3.51
Procrastination × Autonomous motivation										.11	.07	1.19	.12	.09	1.71
Procrastination × Task engagement										.05	.07	.49	.01	.09	.05
Autonomous motivation × Task engagement										.03	.05	.37	−.12	.07	−1.31
Procrastination × Autonomous motivation × Task engagement										−.02	.04	−.12	−.03	.06	−.41
Procrastination^2^ × Autonomous motivation										02	.07	.51	.04	.05	.57
Procrastination^2^ × Task engagement										−.11	.05	−1.81	−.12	.05	−1.84
Procrastination^2^ × Autonomous motivation × Task engagement													.11	.06	2.18*
R^2^	.11			.11			.17			.22			.24		
f^2^	.12			.12			.21			.28			.32		

*Note. Observations* = 213. Gender was coded as 0 = Female, 1 = Male. Education was coded as 1 = College Graduate, 2 = Bachelor Degree, 3 = Master Degree.

*p < .05. **p < .01.

**Table 5:** Multicollinearity assessment.

Predictor Set	Collinearity Statistics	
	Tolerance	VIF
Set 1	0.597	1.674
	0.619	1.614
	0.745	1.343
	0.678	1.476
	
Set 2	0.703	1.423
	0.703	1.423
	0.612	1.634
Set 3	0.924	1.082
	0.924	1.082
	0.856	1.113
Set 4	0.729	1.372
	0.729	1.372
Set 5	0.886	1.129
	0.886	1.129
Set 6	0.715	1.383
	0.715	1.383

To establish an inverted-U-shaped relationship, it's not enough to achieve a notable quadratic effect; it is also essential for the slopes on the left side of the curve to be notably positive, the slopes towards the right side of the curve are significantly negative, as well as the turning point falls inside the range of data [113]. The two-lines test can be used to analyze these circumstances [114]. For a low-moderate range of procrastination, the connection of procrastination with creativity was substantial as well as favorable; however, for a moderate-high range of procrastination, it was significant and negative. The turning point lies inside the data range since the slopes in the two-lines test are calculated from the data. When considered collectively, these findings support Hypothesis 1.

**Fig. 2:**
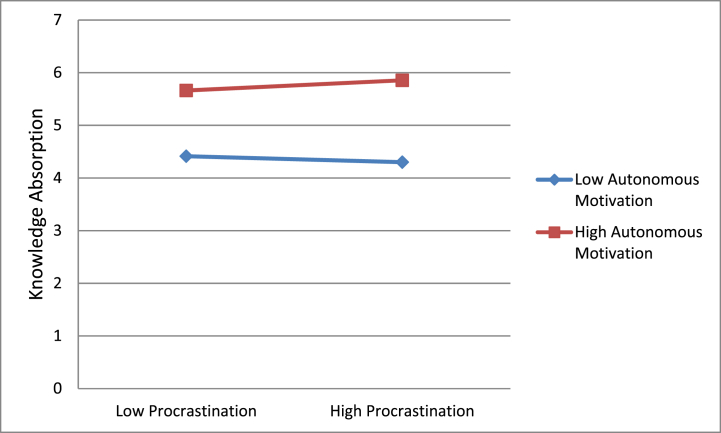
Moderating role of autonomous motivation.

**Fig. 3:**
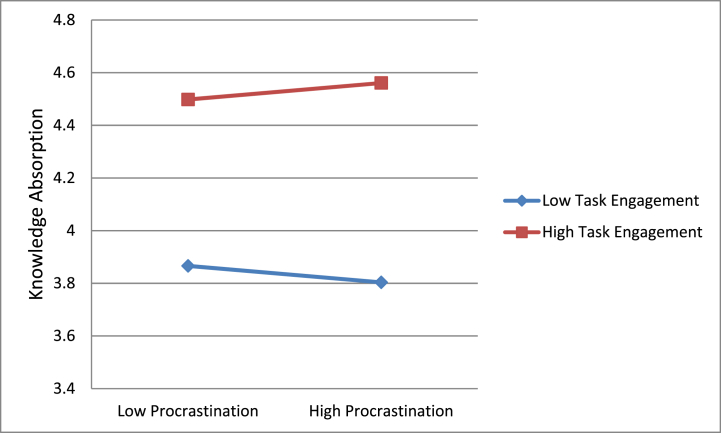
Moderating role of task engagement.

**Fig. 4:**
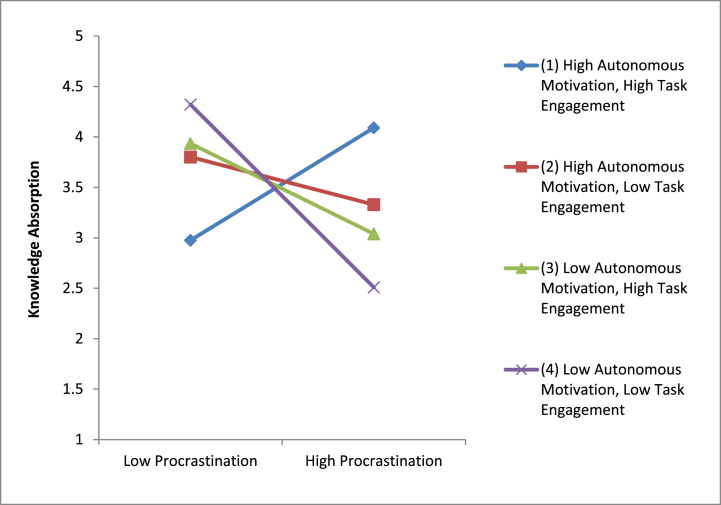
Three-way interaction between procrastination, autonomous motivation, and task engagement.

Following the steps of hierarchical regression, we confirmed the mediation mechanism of knowledge absorption on the correlation of procrastination with creativity. Table 3 provides the outcomes. These findings are indicative of a full mediation of knowledge absorption for the correlation of procrastination with creativity, supporting hypothesis 2 of this research. Additionally, we also confirmed the moderating role of autonomous motivation and task engagement on the correlation that procrastination holds with knowledge engagement. Table 2 provides the moderation outcomes, shown in Fig. 2, Fig. 3. The interaction plots are suggestive of the fact that procrastination had a progressive effect on knowledge absorption when autonomous motivation was also high and low otherwise. Similarly, procrastination had a progressive effect on knowledge absorption as well when task engagement was also high and low otherwise. Table 2 provides a three-way interaction plot also shown in Fig. 4; this plot is suggestive of the fact that procrastination has a positive impact on knowledge absorption only when autonomous motivation and task engagement both were high.

**Fig. 5:**
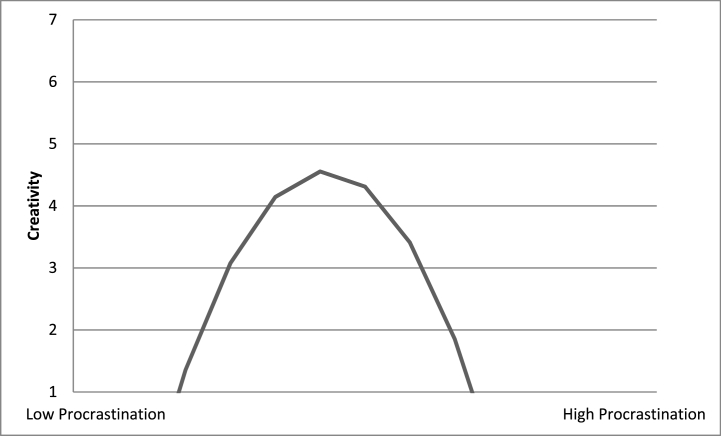
Curvilinear relationship.

**Fig. 6:**
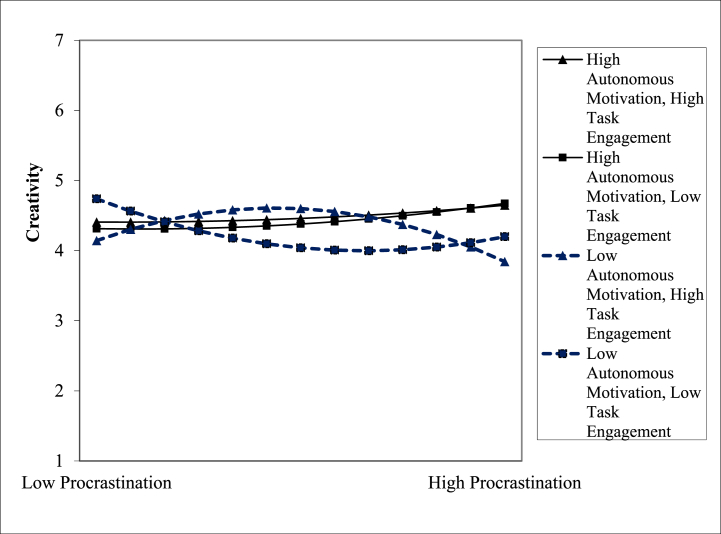
Curvilinear three-way interactions.

Two more models were built to test Hypothesis 3. The linear and quadratic two-way interactions are shown in Step 4, 5 of Table 4, and the quadratic three-way interaction with procrastination squared, the need for creativity, and autonomous motivation is shown in Step 5. When autonomous motivation and/or task engagement are strong, procrastination exhibits an inverted U-shaped association; however, while both autonomous motivation and task engagement are low, procrastination has a negative linear relationship (Fig. 6). The low-low slope is considerably linear but not significantly curved. The high-high curve, however, is not substantially different from the low task engagement-high autonomous motivation curve or the high task engagement-low autonomous motivation curve. These findings are in conformity with Hypothesis 3 in that moderate procrastination fosters creativity when individuals are autonomously driven or held to high standards of task engagement, while it doesn't apply when either of these variables is present.

## 5 General discussion

By triggering the incubation mechanisms of knowledge absorption, this research showed that moderate procrastination has a causal effect on the generation of creative insights. This research demonstrated that as long as employees had strong autonomous drive or high task engagement, their supervisors awarded them better ratings when they procrastinated moderately on their assignments. The quantity of work that workers generated was not in conformity with such a curvilinear link that procrastination exhibits with the caliber of ideas. These findings have significant ramifications for future studies in motivation, creativity, and time management.

### 5.1 Theoretical contributions

The widely held belief that procrastination is an unhealthy habit is contested by this study. It is suggested that if people are autonomously driven or engage in tasks for the evolution of novel insights, moderate procrastination may allow incubation along with encouraging creativity, despite the fact that people may perceive that holding-off on tasks has its consequences. This study disrupts long-held notions about time management by doing this. Investigations previously done on time management skills have highlighted that individuals gain from having the ability to properly prioritize activities, utilize wait periods productively, as well as deadline compliance [115]. These workers may require the ability to postpone the start, advancement, or fulfillment of a job being simultaneously aware that there could be a drawback to that activity in order to produce ideas that are innovative and beneficial. Research indicates that while these abilities are crucial for productivity, they don't necessarily play a favorable role in creativity. Employees may need the capacity to put off starting, moving on with, or finishing a task while still realizing any possible downsides in order to come up with unique and worthwhile thoughts.

The investigation sheds light on the function of incubation in the development of creativity. Available research suggests that allowing time for insights to develop might occasionally increase creativity while, in other instances can also decrease it [116]. It is discovered that moderate procrastination solely boosts the end product's creativity, despite the fact that both moderate as well as extreme procrastination involve incubation periods. Workers can put in continual efforts in structural reforming of the issue while stimulating innovative perspectives during moderate procrastination because significant progress occurs outside of the task's initiation and completion timelines, leading to a creative end. In contrast, when workers procrastinate a lot and make most of their progress toward the job deadline, they could feel pressured to do it as soon as possible. This may hinder their capacity to reconstruct issues and draw upon fresh information, eventually producing less original results. The findings from the investigation deepen existing knowledge of curvilinear interactions by demonstrating the importance of progress timing for efficient incubation [36,86]. This research shows that a modest amount of procrastination, which strikes a balance between starting right away and racing to meet a deadline, is best for effective incubation.

Furthermore, this research advances our knowledge of autonomous motivation. However, empirical examination has shown that intrinsic motivation only accounts for the extent of smaller variations in procrastination [28]. During the field study, it was also found no significant correlation between the two variables, despite previous research frequently viewing intrinsic motivation as a cure for procrastination. When intrinsic motivation may not always stop employees from delaying, the theoretical viewpoint and empirical data from this study imply that autonomous motivation can influence how they direct all focus being engaged in procrastination. This emphasizes a parallel function for autonomous motivation in the creative process and emphasizes the significance of looking at how autonomous motivation affects attention during off-task activities in addition to behavior during specific tasks. The outcomes from the current research emphasize the point that autonomous motivation may influence behavior in addition to just producing it. Furthermore, it was found that task engagement may make up for and encourage creativity under mild procrastination in the absence of autonomous motivation. This shows that, rather than serving as a prerequisite for autonomous driving, task engagement could also be viewed as a replacement for it.

## 6 Limitations and future directions

It is critical to recognize the limits of this study, which must be taken into account while analyzing the outcomes. The complete model must be evaluated in future studies because this specific study only examined the mediators and moderators in individual trials. Second, this research did not specifically explore how the reasons for procrastination or the choices made for activities to engage in while procrastinating may affect its outcomes. Is it better for workers to put off tasks when they put them off because they are unhappy with their first ideas as opposed to when they fail to exercise self-control or opt for intense working conditions? Does procrastinating employees' use of meaningless tasks free up cognitive resources [63] while reducing any costs of attention residue [117]? Additionally, a contrasting impact that makes any postponed job further unpleasant [20] may be produced if the alternative activity is extremely autonomously motivated. However, if the substitute activity is reasonably engaging, it could inspire excitement that spills over into the postponed work, either directly stimulating creativity [118] or by stoking the emotional uncertainty that frequently accompanies procrastination in addition to guilt and worry. It would be beneficial to conduct further study on the function of various procrastination-related motivations and the activities procrastinators engage in.

Thirdly, our results showed that the curvilinear impact that procrastination inflicts upon creativity was mediated by knowledge absorption; however, this raises concerns about potential additional pathways. The incubation process of selective forgetting, which entails tossing out first answers that may be imitative or impracticable [98], may be one cause. It's also conceivable that this study did not fully account for the spectrum of conscious and unconscious incubation processes that promote creativity in measurements. Additionally, procrastination could affect creativity via processes other than incubation. For instance, mild procrastination may help in idea selection by allowing people to consider the standards for judging originality and usefulness and to get over objections to innovative concepts [119]. This may help to explain why task engagement has a greater facilitative moderating effect than an autonomous incentive, which can increase the focus on inventive but perhaps ineffective solutions [86]. However, it's also likely that creativity declines when autonomous drive approaches zero.

Fourthly, the research raises the possibility that mild procrastination may enhance creativity, but there are still numerous open issues and research constraints that need to be resolved in future investigations. Establish standards for determining the point at which procrastination starts to spur creative output. Exploring the ideal amount of task exposure or task progress before procrastination can become beneficial for creativity. Examine the function of various motivations triggering procrastination along with the details of engagements while procrastinating. Future research might further look at the dynamics of task engagement and procrastination as they relate to creativity over time, as well as if the absence of emotional influences was a result of the measuring techniques. Taking care of these problems will help us comprehend the intricate connection between procrastination and creativity.

Sixth, while this research focused on the correlation of procrastination with creativity within a personal capacity, understanding this relationship on a community level might be useful [120]. In scenarios where the creative process for a particular project depends on sequential dependency, one team member's tardiness may limit the originality of the group because it may impede the work of other members [64]. On the other side, it's conceivable that opting to procrastinate by any singular entity within a team may promote procrastination in other members in moderation, in turn supporting incubation, especially if individuals are genuinely driven or engaged in creative activities. It would also be helpful to look at the social processes that explain how moderate procrastination fosters creativity. For instance, delaying could increase the likelihood that workers would receive unexpected ideas and helpful criticism through weak ties [53]. Finally, a study into procrastination's organizational predecessors is necessary in addition to its effects. What part do deadlines play, plus which deadlines seem to encourage a predisposition for mild procrastination, for instance? The research presents a conceptual framework for regulating procrastination while evaluating its degree in laboratory and online environments that might assist in the assessment of such crucial concerns. Previous studies have faced difficulties researching procrastination experimentally.

## 7 Practical implications and conclusion

In the office, procrastination is frequent and may cause a lot of stress. In light of the fact that it is generally considered to be a lack of self-regulation, research indicates that it can cause negative emotional states such as fear, shame, and guilt [121]. These unfavorable emotions may make things worse and cause a “depression spiral” [28]. Procrastination can be difficult to eradicate, though entirely. Therefore, there are some situations when it could be advantageous to employ it creatively. For instance, it may be desirable to moderately postpone the task's start, advancement, or finish when personnel are autonomously driven or require fresh and helpful ideas. Additionally, managers and leaders might promote creative procrastination by initiating any innovative practice by posing a challenge but delaying looking for suggestions/solutions. Nevertheless, it is crucial to prevent procrastination from getting in the way of doing the task at hand. In order to prevent long-term procrastination, it may be helpful to educate employees about the benefits of delaying rather than giving them an excuse to do so [121,122].

Conclusively, procrastination has been traditionally seen as a behavior that organizations should eradicate. With this research, we have shown that in the business world, positive benefits of procrastination can be achieved, and managers can capitalize on the benefits of procrastination for the creative potential of employees. This is specifically beneficial for organizations when the culture of the organization is continuously changing to a less rigid culture [123,124]. Although it might be difficult for many employees, procrastinating can be good for creativity. Procrastinators could come up with superior concepts. If one is going to procrastinate, one may as well be brilliant at it, according to Margaret Atwood, who said that she had racked up years and years of it [6,20]. In his journal, Leonardo da Vinci expressed remorse and questioned whether anything ever got accomplished. However, researchers contend that artistic procrastination is appropriate if it allows Leonardo to put off finishing a few small commissions while he works on bigger ones [27].

Additionally, it has also been found that business organizations can get benefits from active procrastination, as it can improve decision-making, time management, motivation, and utilization of available resources [125,126]. Procrastination can help individuals gather relevant information and evidence-building needed for better decision-making and task completion [127]. Examples can be found in the business world, where organizations have benefited from individual procrastination. The technology industry has been found to have benefited from procrastination by improved creativity. For more divergent thinking and creative ideas to come, Steve Jobs has been found to delay his immediate tasks [128]. For challenging and critical tasks, Bill Gates has been found to hire people who are considered to be lazy as these lazy individuals come up with more creative ideas [129]. The only people who would be critical of him for it is those who are wholly enslaved by the present religion of productive mediocrity.

## Ethics statement

This study was approved by the Ethical Committee- The University of Chenab; Ref: 03BE23.

## Funding statement

This research is partially funded by Princess Nourah bint Abdulrahman University Researchers Supporting Project number (PNURSP2023R4), Princess Nourah bint Abdulrahman University, Riyadh, Saudi Arabia.

## Author contribution statement

Ahmad Adeel: Conceived and designed the experiments; Analyzed and interpreted the data; Contributed reagents, materials, analysis tools or data; Wrote the paper.

Samad Sarminah: Contributed reagents, materials, analysis tools or data.

Li Jie: Conceived and designed the experiments; Performed the experiments; Analyzed and interpreted the data.

Kee Mui Hung Daisy: Analyzed and interpreted the data; Performed the experiments.

Yahya Qasim Daghriri: Contributed reagents, materials, analysis tools or data.

Rsha Ali Alghafes: Contributed reagents, materials, analysis tools or data.

## Research involving human participants and/or animals

Data were collected from humans only after obtaining formal approval, which is also mentioned in the methodology section, and we confirm that no humans were harmed during the data collection process.

## Informed consent

Article submitted with consent of all researchers, our names can be used as authors of this article.

## Submission declaration and verification

Submission of an article implies that the work described has not been published previously.

## Use of inclusive language

The Authors ensure that writing is free from bias, stereotypes, slang, reference to dominant culture and/or cultural assumptions.

## Data availability statement

Data will be made available on request.

## Declaration of generative AI and AI-assisted technologies in the writing process

During the revision of this research article the author(s) used Grammarly in order to improve language quality. After using this tool/service, the author(s) reviewed and edited the content as needed and take(s) full responsibility for the content of the publication.

## Declaration of competing interest

The authors declare that they have no known competing financial interests or personal relationships that could have appeared to influence the work reported in this paper.
