# Supplementary material for: When procrastination pays off: Role of knowledge sharing ability, autonomous motivation, and task involvement for employee creativity
Source: Heliyon. 2023 Sep 11;9(10):e19398. doi: 10.1016/j.heliyon.2023.e19398 (PMC10520733; doi:10.1016/j.heliyon.2023.e19398)
Supplement: Multimedia component 1 [file mmc1.docx]

**Scale Items**

| **Knowledge Absorption** |
| --- |
| I may promptly judge how more useful the new information and knowledge is than the existing ones. |
| I may accept the task-related information and knowledge well. |
| I am good at finding out the required information and knowledge |
| I may organize important data for the future reference well |
|  |
| **Autonomous Motivation** |
| Why do you absorb knowledge? |
| ...because I enjoy it |
| ...because I like it |
| ...because I find it personally satisfying |
| ...because I think it is an important part of my job |
|  |
| **Task Engagement** |
| I exerted my full effort in the task |
| I try my hardest to perform well on the task |
| I strive as hard as I can to complete the task |
| I felt energetic working on the task |
| I am excited working on the task |
| I feel positive working on the task |
| I concentrated completely on the task |
| I am absorbed by the task |
| I devote a lot of attention on the task |
|  |
| **Employee Creativity** |
| How often does this employee |
| …creating new ideas for improvements |
| …searching out new working methods, techniques, or instruments |
| …generating original solutions to problems |
|  |
| **Intrinsic Motivation** |
| Why are you motivated to do your work? |
| Because I enjoy the work itself |
| Because it’s fun |
| Because I find the work engaging |
| Because I enjoy it |
| **Extrinsic Motivation** |
| Why are you motivated to do your work? |
| Because I need to pay my bills |
| Because I need to earn money |
| Because I have to |
| Because I need the income |
| Because I want to avoid feeling guilty |
| Because I’ll feel bad about myself if I don’t |
| Because I want to avoid looking bad |
| Because I’ll feel ashamed if I don’t |
| Because I think it’s important |
| Because I don’t want to cause harm |
| Because it’s satisfying |
| Because I want to do a good job |
